# Generation of endogenously tagged E-cadherin cells using gene editing via non-homologous end joining

**DOI:** 10.1016/j.xpro.2023.102305

**Published:** 2023-05-12

**Authors:** Natalie Rimmer, Ching-Yeu Liang, Ricardo Coelho, Monica Nunez Lopez, Francis Jacob

**Affiliations:** 1 Ovarian Cancer Research, Department of Biomedicine, University Hospital Basel and University of Basel, Basel 4031, Switzerland; 2 Hospital for Women, University Hospital Basel, Basel 4031, Switzerland

**Keywords:** Cell Culture, Flow Cytometry/Mass Cytometry, Cell Separation/Fractionation, Gene Expression, CRISPR

## Abstract

We provide a protocol using non-homologous end joining to integrate an oligonucleotide sequence of a fluorescence protein at the *CDH1* locus encoding for the epithelial glycoprotein E-cadherin. We describe steps for implementing the CRISPR-Cas9-mediated knock-in procedure by transfecting a cancer cell line with a pool of plasmids. The EGFP-tagged cells are traced by fluorescence-activated cell sorting and validated on DNA and protein levels. The protocol is flexible and can be applied in principle to any protein expressed in a cell line.

For complete details on the use and execution of this protocol, please refer to Cumin et al. (2022).^1^

## Highlights

• CRISPR-Cas9-mediated gene editing in a human ovarian cancer cell line
• Non-homologous end joining is a suitable mechanism for tagging proteins
• Knock-in EGFP at the C-terminus of E-cadherin

## Abstract (editor highlights)

Publisher’s note: Undertaking any experimental protocol requires adherence to local institutional guidelines for laboratory safety and ethics.

## Graphical abstract

**Figure:**
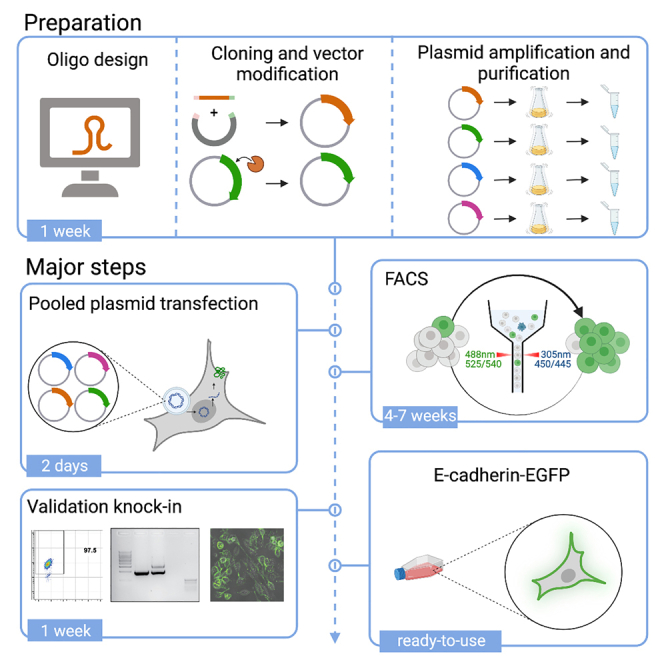

## Before you begin

**Figure 1:**
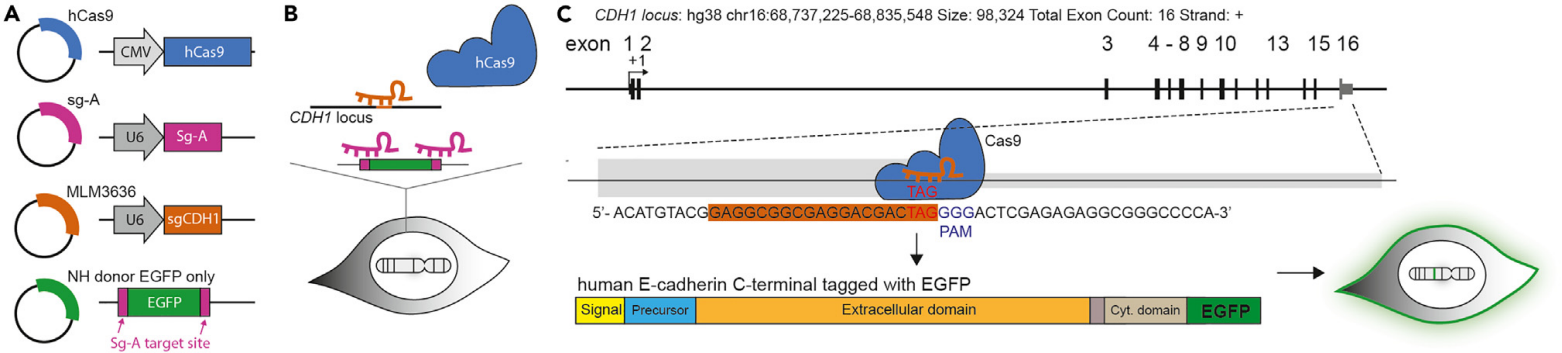
Schematic representation of the gene editing strategy to tag endogenous E-cadherin at the C-terminus with Enhanced Green Fluorescence Protein (A) Plasmids necessary for the generation of a knock-in cell line. (B) Principle of sgRNA binding and Cas9 activity upon transient delivery of four plasmids. (C) Targeted *CDH1* locus resulting in endogenous E-cadherin C-terminally tagged with EGFP.

Gene-editing using the CRISPR-Cas9 system is nowadays widely applied in human cells. Upon Cas9-induced DNA double-strand breaks, cells rely on a non-homologous end joining (NHEJ) repair pathway prone to efficiently induce insertion or deletion (indel) mutations mostly resulting in gene knockouts at the targeted genomic site.^2^ In regard to gene knock-ins, homology-directed repair (HDR) is the preferred way to integrate large single- and double-stranded oligonucleotide sequences, however, it seems to be difficult to generate due to low efficiency.^3^^,^^4^ In contrast to HDR generated knock-ins, NHEJ has been suggested to be more efficient for generation of knock-ins as it does not rely on a specific cell cycle and does not require regions of homology. Despite its believed error-prone character, NHEJ has been recently shown to accurately ligate DNA ends without indels.^5^^,^^6^ In order to overcome limitations using HDR, here, we take advantage of NHEJ for integrating reporter genes to tag cell surface proteins. In this protocol, we provide a detailed experimental section on developing a plasmid-based knock-in in the human ovarian cancer cell line BG1 using the CRISPR-Cas9 system. This approach builds on a previously reported knock-in strategy,^3^ here, further adapted to generate endogenously tagged proteins, exemplified for the transmembrane protein E-cadherin. The concept of this knock-in strategy is the combination of efficient and precise gene editing using NHEJ in human cancer cells as demonstrated in a previous study.^3^ The sgRNA is designed to target the E-cadherin-encoding translation stop site as close as possible. In principle, we provide a set of plasmids (n = 4) necessary to integrate the EGFP-encoding sequence as double-stranded DNA at the genomic locus of *CDH1* (Figure 1A). The transient transfection of cancer cells results in high expression of hCas9 together with two sgRNAs controlled by the U6 promoter. The first sgRNA (sgCDH1) binds to the genomic locus while the second sgRNA (Sg-A) targets the flanking sites of the fourth plasmid encoding EGFP (Figure 1B). The released *EGFP* will then be integrated into *CDH1* via NHEJ (Figure 1C). If integrated in-frame and translated into a fusion protein, the EGFP signal will allow direct assessment of the knock-in efficiency by flow cytometry and further enrichment of those cells.

### Preparation 1: Modification of double cut NH-donor resulting in “double-cut EGFP only”

**Timing: 6 days**

The internal ribosome entry site (IRES) is a *cis*-acting RNA sequence directly recruiting ribosomes to an internal position of the transcribed mRNA in a cap-independent manner to initiate translation.^7^ The original plasmid double cut non-homology (NH) donor encodes both, the IRES and EGFP flanked by two sgRNA recognition sites (Sg-A) to introduce DNA cleavage for the desired integration,^3^ allowing separate expression of a target gene and EGFP. In order to fuse EGFP C-terminally to E-cadherin, the IRES has to be removed from the original plasmid. The process is described in more detail below.

1. Day 1–2: Design and order PCR primers for removing the IRES from double cut NH-donor by NEBaseChanger (https://nebasechanger.neb.com/).

**Table:** 

Name	5′ – 3′
EGFP no IRES_F	ATG GTG AGC AAG GGC GAG
EGFP no IRES_R	CAA TTG CCG GTG ATG CGG

2. Day 3: Perform the protocol of the NEB Q5® Site-Directed Mutagenesis Kit (LINK).
  a. Site-directed mutagenesis PCR.
    i. Dilute the plasmid of double cut NH-donor in ddH_2_O to 20 ng/μL.
    ii. Prepare the PCR reaction as shown in Table 1, then mix well and spin down briefly.

**Table 1:** PCR reaction

Reagent	Volume in μL
Q5 Hot Start High-Fidelity 2× Master Mix	12.5
EGFR no IRES_F 10 μM	1.25
EGFR no IRES_R 10 μM	1.25
Double cut NH-donor plasmid DNA (20 ng/μL)	1
ddH_2_O	up to 25
    iii. Use the following Thermocycler programme for annealing (Table 2):

**Table 2:** PCR programme

Step	Temperature	Time	Cycles
Initial denaturation	98°C	30 s	1
Amplification	98°C	10 s	25
	63°C	30 s	
	72°C	4 min	
Final extension	72°C	2 min	1
Hold	4°C
  b. Kinase-Ligase-*DpnI* (KLD) reaction.
    i. Prepare the digestion reaction as shown in Table 3, mix well and spin down.

**Table 3:** Kinase-Ligase reaction

Reagent	Volume in μL
PCR product	1
2× KLD Reaction buffer	5
10× KLD enzyme mix	1
ddH_2_O	3
    ii. Incubate at 20°C–22°C for 5 min.
  c. Heatshock transformation into competent cells.
    i. Thaw competent cells (DH5-alpha) on ice for 30 min.
    ii. Add 100 μL of the DH5-alpha to the ligation reaction.
    iii. Mix gently and place on ice for 30 min.
    iv. Incubate the reactions at 42°C for 45 s.
    v. Place immediately on ice for 2 min.
    vi. Add 500 μL of 2×YT Broth (20°C–22°C), then incubate at 37°C for 1 h with low agitation (300 rpm) on an orbital shaking thermoblock. During this time, pre-warm LB-Carb-plates (LB-agar plates containing 100 μg/mL of carbenicillin) at 37°C.
    vii. Plate out 100 μL of the transformed cells on the pre-warmed LB-Carb-plate.
    viii. Incubate 16 h at 37°C.

***Alternatives:*** The 2×YT is a rich medium used in our laboratory, however, LB broth or Terrific Broth are also suitable for the transformation procedure with DH5-alpha. Instead of using DH5-alpha other competent cells such as Stbl3 are also possible.

3. Day 4: Pick up a colony from the plate using a pipet tip, and place it into a 14 mL round-bottom tube containing 3 mL of LB-Carb-broth (LB broth containing 100 μg/mL of carbenicillin).
  a. Repeat this step for 5–10 colonies.
  b. Incubate at 37°C shaking at 200 rpm for 16 h overnight.
4. Day 5: Plasmid preparation, Sanger DNA sequencing and glycerol stocks.
  a. Perform a plasmid preparation using the ZR Plasmid Miniprep - Classic Kit according to the manufacturer’s protocol (LINK). For this purpose, use 2 mL of the overnight culture.
  b. Measure the concentration and purity to prepare the plasmid DNA for Sanger DNA sequencing.
  c. In our case, selected clones were sequenced using the Sanger DNA sequencing service provided by Microsynth AG, Switzerland.
    i. For this purpose, a total of 1.2 μg of plasmid in 12 μL of ddH_2_O was shipped and sequenced with EGFP-C-for and EGFP-N-rev.
    ii. In parallel, prepare a glycerol stock using 500 μL of the remaining overnight culture and mix it with 500 μL of 50% (w/v) glycerol solution (50% in ddH_2_O, filtered 0.2 μm).
5. Day 6: Confirm absence of the IRES element by analyzing Sanger DNA sequencing results.

### Preparation 2: Design and cloning of sgRNA targeting the *CDH1* locus

**Timing: 6 days**

This section provides details on how to design and clone an sgRNA that will be used for inserting the EGFP-encoding DNA sequence into the *CDH1* locus. An sgRNA sequence for gene-editing of a desired genomic location is a 20 nucleotide sequence (target sequence), followed by the protospacer adjacent motif (PAM) NGG (for details also see Figure 1C) **(**troubleshooting 1).

1. Day 0: Design and ordering of oligonucleotides.
  a. If you do not already have one, create an account at www.benchling.com.
  b. Log in and start a new project.
  c. Import the human *CDH1 sequence* and annotate the protein-encoding sequence if not already present.
  d. The imported *CDH1* sequence can be searched for sgRNAs at the C-terminal protein coding sequence.
    i. For this purpose, select the CRISPR button on the right side of the website.
    ii. You then need to “Design and analyze guides” and select a single guide, 20 bp length, the corresponding human genome, and the “NGG” PAM.
      ***Note:*** The described settings are suitable for the current experimental setup using the Cas9 which has been derived from a human codon-optimized *Streptococcus pyogenes* Cas9 (hSpCas9).
  e. Highlight the *CDH1 exon* 16 target region in the “Sequence map” tab.
  f. Next, use the “Design *CRISPR*” tab and click on the “+” symbol in order to obtain possible sgRNAs. We have selected the sgRNA targeting (5′-GAG GCG GCG AGG ACG ACT AG-3′) upstream of the translation stop site of E-cadherin with an “on-target” and “off-target” score of 18.7 and 91.5, respectively.
    ***Note:*** If there are other proteins that are of interest, you may also consider selecting the sgRNAs with high “on-target”^8^ and “off-target”^9^ scores using benchling (troubleshooting 2).
    ***Alternatives:*** There are various online tools for sgRNA search and design. *In silico* platforms such as CRISPick (https://portals.broadinstitute.org/gppx/crispick/public) or ChopChop (http://chopchop.cbu.uib.no/) are serving the purpose.

2. Day 1: Cloning:
  a. Annealing of oligonucleotides:
    i. Dissolve the lyophilized oligonucleotides (desalt purification) in nuclease-free ddH_2_O in order to prepare a solution of 100 μM.
    ii. Prepare the annealing reaction by diluting the oligonucleotides in a ratio of 1:20 in ddH_2_O as shown in the following table, then mix well and spin down briefly:

**Table:** 

Reagent	Annealing reaction (in μL)	Control 1 (in μL)	Control 2 (in μL)
sgCDH1_F1 (100 μM)	5	5	–
sgCDH1_R1 (100 μM)	5	–	5
ddH_2_O	90	95	95
TOTAL VOLUME	100	100	100
    iii. Use the following Thermocycler programme for annealing:

**Table:** 

Steps	Temperature	Time	Cycles
Denaturation	95°C	5 min	1
Decreasing temperature gradient	94°C ΔT–1°C	1:45 min / step	71
Hold	16°C	Forever	–
    iv. Visualize annealed oligonucleotides with a 2% (w/v) agarose gel (e.g., 2 g of agarose in 100 mL of 1× TAE buffer (diluted from 50× TAE buffer with ddH_2_O) (Figure 2A).

**Figure 2:**
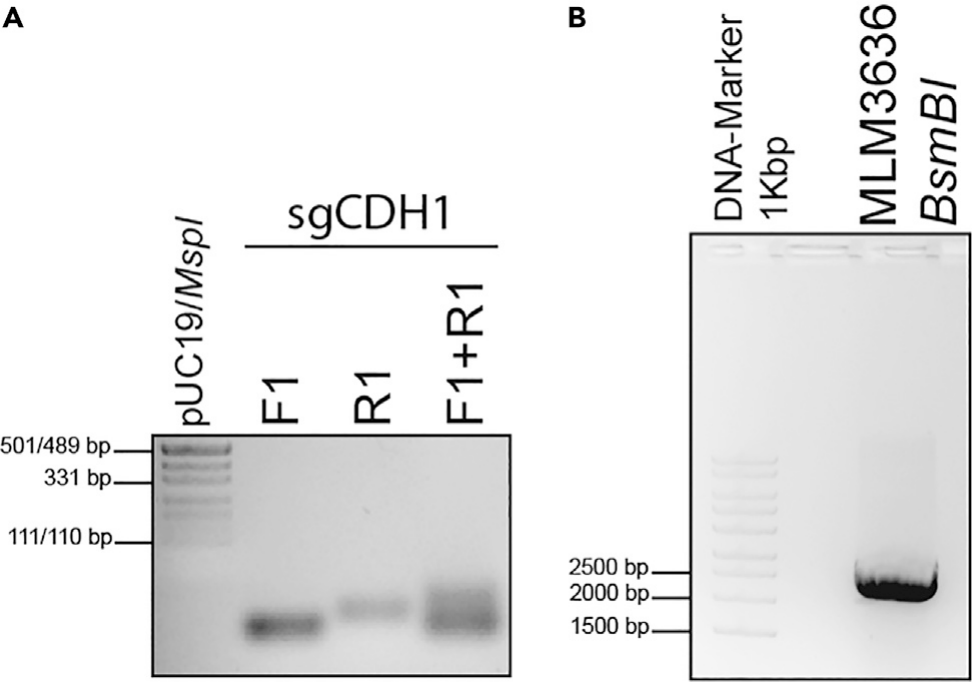
Cloning of sgCDH1 oligonucleotides into MLM3636 plasmid (A) Representative agarose gel showing single-strand and aligned (sgCDH1) oligonucleotides. (B) Agarose gel visualizing *BsmBI*-digested plasmid MLM3636.
  b. Vector digestion:
    i. Prepare the digestion reaction of the plasmid MLM3636 using the restriction endonuclease *BsmBI* (Table 4).

**Table 4:** Digestion reaction

Reagent	Amount in μL
NEB Buffer r3.1	5 (1×)
DNA (MLM3636) 1–2 μg	×
*BsmBI-*v2	1–2 (10–20 Units)
ddH_2_O	up to 50
    ii. Use the following programme for the digestion:

**Table:** 

Steps	Temperature	Time
Digestion	55°C	2 h
Enzyme inactivation	80°C	20 min
Hold	16°C	Forever
    iii. Run a 1% (w/v) agarose gel and cut out the band at a size of 2′253 bp (Figure 2B).
    iv. Perform agarose gel purification using the Wizard® SV Gel and PCR Clean-Up System kit according to the manufacturer’s instructions (LINK).
      ***Alternatives:*** Apart from the above-mentioned kit for agarose gel purification other equivalent kits or protocols can be used (e.g. Zymoclean™ Gel DNA Recovery Kit, ZYMO RESEARCH).
      **Pause point:** The annealed oligonucleotides as well as the digested and purified plasmid can be stored at −20°C for long-term storage.
  c. Ligation of annealed oligonucleotides with purified digested vector:
    i. Dilute the annealed oligonucleotides in a ratio of 1:200 (e.g., 5 μL of sgCDH1_F1+R1 and 995 μL of ddH_2_O).
    ii. Prepare the ligation reaction as shown in Table 5 and add a control replacing the annealed oligonucleotides with the same volume of ddH_2_O. Incubate the ligations for 16 h overnight at 4°C.

**Table 5:** Ligation reaction

Reagent	Amount in μL
MLM3636 (digested + purified) 100 ng	×
sgCDH1_F1+R1 (diluted 1:200)	2
2× Rapid Ligation Buffer	10
T4 DNA Ligase	1
ddH_2_O	up to 20

3. Day 2: Heatshock transformation into competent cells as described above.
  a. Plate out 100 μL of the transformed cells on the pre-warmed LB-Carb-plate.
  b. Incubate for 16 h overnight at 37°C.
4. Day 3: Pick colonies and prepare overnight cultures.
  a. Prepare the mastermix for a colony PCR (Table 6) using human U6 as forward and the reverse sgRNA as reverse primer:

**Table 6:** PCR reaction master mix

Reagent	Amount per reaction in μL
DNA template	×
MyTaq DNA Polymerase (5U/μL)	0.15
human_U6_F (forward) (10 μM)	0.3
sgCDH1_R1 (reverse) (10 μM)	0.3
5× MyTaq Reaction Buffer	3
ddH_2_O	up to 15
  b. Pick up a colony with a pipet tip, dip it into the PCR tube containing the prepared mastermix and then place the tip into a liquid overnight culture (14 mL round-bottom tube containing 3 mL of LB-Carb-broth).
    i. Repeat this step for ∼10 colonies.
  c. The colony PCR is performed with the following parameters (Table 7):

**Table 7:** PCR cycling conditions

Steps	Temperature in °C	Time	Cycles
Initial Denaturation	94	4 min	1
Denaturation	94	30 s	32 cycles
Annealing	55	15 s	
Extension	72	30 s	
Final extension	72	5 min	1
Hold	16	Forever
  d. Visualize the PCR result with a 1.5% (w/v) agarose gel (Figure 3).

**Figure 3:**
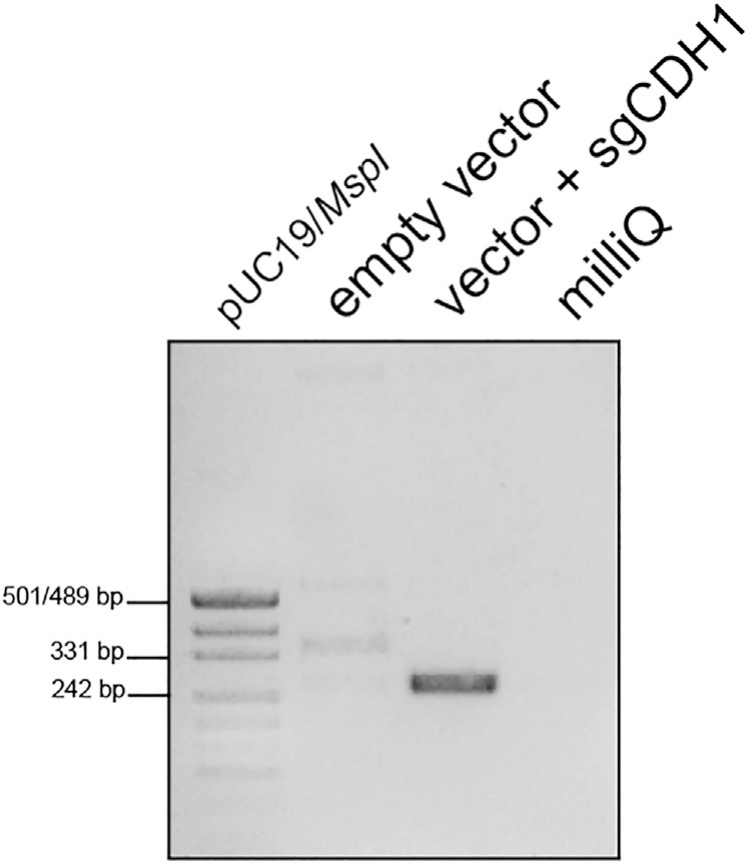
Representative agarose gel separating colony PCR-derived amplicons The expected product size is 274 bp as visible for “vector + sgCDH1.”
  e. The overnight cultures of the positive colonies are incubated at 37°C shaking at 200 rpm for 16 h overnight.
5. Day 4: Plasmid preparation, Sanger DNA sequencing and glycerol stocks.
  a. Perform a plasmid preparation using the ZR Plasmid Miniprep - Classic Kit according to the manufacturer’s protocol (LINK). For this purpose, use 2 mL of the overnight culture.
  b. Measure the concentration and purity and prepare the plasmid DNA for Sanger DNA sequencing as described above.
    i. In our case, selected clones were shipped along with 10 μM of human_U6_F primer in a separate tube.
  c. In parallel, prepare a glycerol stock as described above.
6. Day 5: Analyze the DNA sequencing results.
  a. We usually use 4Peaks for visualization of .ab1 sequencing files (Figure 4).

**Figure 4:**
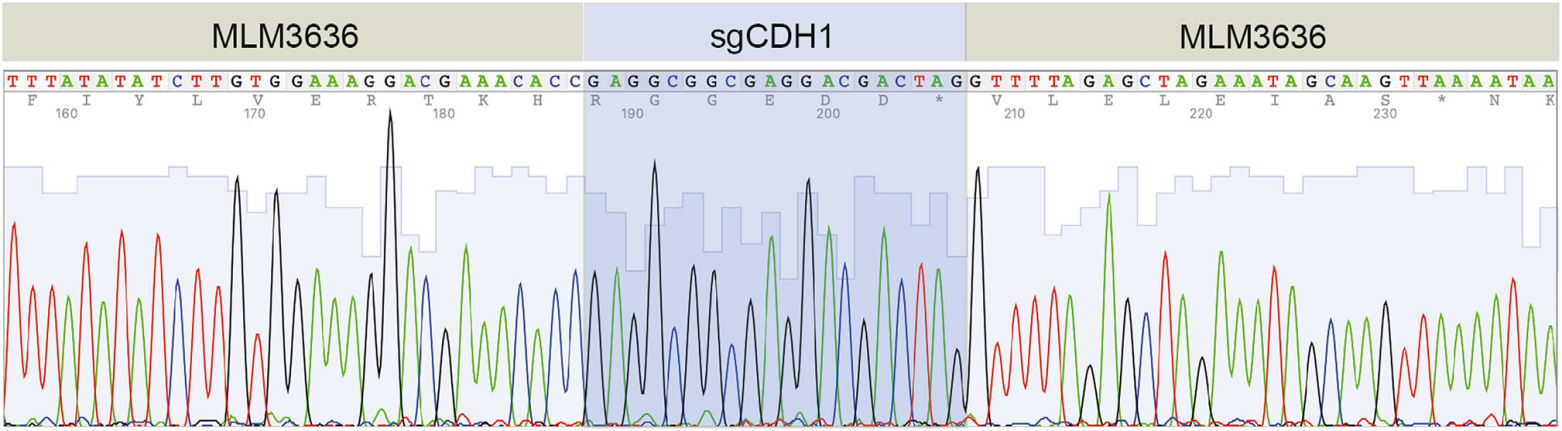
Sanger DNA sequencing of sgCDH1 ligation into MLM3636 plasmid
  b. If the DNA sequence confirms successful insertion of the desired oligonucleotide, either prepare more plasmid (mini- or midiprep) or directly continue with transient transfection of the desired cell line (BG1) as described below. This construct is hereinafter referred to as “sgCDH1”.

**Pause point:** The plasmid DNA can be stored at −20°C for long-term storage.

### Preparation 3: Preparation of plasmids

**Timing: 4 days (not including the ordering)**

1. Day 0: Order the necessary plasmids listed in the key resources table below.
2. Day 1: Streak the received bacterial stab cultures onto an LB-Carb-plate in order to isolate single colonies. Keep the inoculated LB-Carb-plates for 16 h overnight at 37°C.
3. Day 2: Pick up a single colony and inoculate an overnight liquid culture (3 mL of LB-Carb-broth) for 16 h at 37°C shaking with 200 rpm.
4. Day 3: Perform a plasmid preparation to purify the plasmid DNA using the ZR Plasmid Miniprep - Classic Kit according to the manufacturer’s protocol (LINK). Use the remaining overnight culture to prepare a glycerol stock.

***Optional:*** If a higher quantity of plasmid DNA is needed, start a new overnight culture for an enlarged plasmid purification, i.e. inoculate 150 mL of LB-Carb-broth with 100 μL of the existing culture and incubate at 37°C, 200 rpm, 16 h overnight. On the next day, perform a plasmid preparation using the NucleoBond® Xtra Midi Plus kit according to the manufacturer’s protocol (LINK).

**Pause point:** The plasmid DNA can be stored at −20°C for long-term storage. Glycerol stocks should be kept at −80°C for long-term storage.

**CRITICAL:** It is suggested to obtain high concentration (∼500 ng/μL) and purity of all plasmids in order to increase the efficiency of transient transfections.

## Key resources table

**Table:** 

REAGENT or RESOURCE	SOURCE	IDENTIFIER
**Antibodies**		

Rabbit monoclonal anti-E-Cadherin (24E10) (Western blot 1:1000; Immunofluorescence 1:400)	Cell Signaling Technology	Cat# 3195; RRID: AB_2291471
Rabbit polyclonal anti-GFP (Western blot 1:5000)	GeneTex	Cat# GTX113617; RRID: AB_1950371
Mouse monoclonal GFP (B-2) (Immunofluorescence 1:400)	Santa Cruz	Cat# sc-9996
Rabbit monoclonal anti-alpha/beta-Tubulin (Western blot 1:2000)	Cell Signaling Technology	Cat# 2148; RRID: AB_2288042
Anti-rabbit IgG HRP-linked Antibody (Western blot 1:10000)	Cell Signaling Technology	Cat# 7074S
Goat anti-Mouse IgG (H + L) Alexa Fluor™ 488 (Immunofluorescence 1:500)	Invitrogen	Cat# A-11001
Anti-rabbit IgG Fab2 Alexa Fluor® 647 (Immunofluorescence 1:500)	Cell Signaling Technology	Cat #4414S

**Biological samples**		

Subcloning Efficiency™ DH5 alpha Competent Cells	Thermo Fisher Scientific	Cat# 18265017

**Commercially available plasmids**		

hCas9	Addgene	Cat# 41815
hCas9_D10A	Addgene	Cat# 41816
MLM3636	Addgene	Cat# 43860
Double cut NH-donor	Addgene	Cat# 83576
Sg-A	Addgene	Cat# 83807
pEGFP-N1∗	Addgene	Cat# 6085-1

**Chemicals, peptides, and recombinant proteins**		

Carbenicillin disodium salt	Carl Roth	Cat# 6344.2
Glycerol	Sigma	Cat# G5516
LB Agar (Lennox)	Carl Roth	Cat# X965.1
LB Broth (Lennox)	Carl Roth	Cat# X964.1
2×YT Broth	Carl Roth	Cat# X966.1
ROTIPHORESE® 50× TAE Buffer	Carl Roth	Cat# CL86.1
Agarose, LE, analytical grade	Promega	Cat# V3125
Dimethyl sulfoxide (DMSO)	Carl Roth	Cat# 4720.1
Trizma® hydrochloride	Sigma	Cat# T3253
Bromophenol blue	Sigma	Cat# B0126
DL-Dithiothreitol	Sigma	Cat# D0632
Albumin fraction V	Carl Roth	Cat# 8076.4
Formalin 4%, gepuffert	Formafix AG	
Triton™ X-100	Sigma	Cat# T-8787
Tween® 20	Sigma	Cat# P1379
SDS	Carl Roth	Cat# 0183.1
Sodium chloride	Carl Roth	Cat# 3957.1
Trizma® base	Sigma	Cat# T1503
Glycine	Sigma	Cat# G7126
30% Acrylamide/Bis Solution 37.5:1	Bio-Rad	Cat# 1610158
TEMED	Bio-Rad	Cat# 161-0801
Ammonium persulfate	Sigma	Cat# A3678
Methanol	Carl Roth	Cat# 8388.6
2-Propanol	Carl Roth	Cat# CP41.3
Hydrochloric acid 37%	Carl Roth	Cat# 9277.1
DAPI	BioLegend	Cat# 422801
ProLong® Gold Antifade Reagent with DAPI	Cell Signaling Technology	Cat# 8961

**Critical commercial assays**		

Wizard® SV Gel and PCR Clean-Up System	Promega	Cat# A9281
ZR Plasmid Miniprep™- Classic Kit	Zymo Research	Cat# D4016
Q5® Site-Directed Mutagenesis Kit	New England Biolabs	Cat# E0552S
NucleoBond® Xtra Midi Plus kit	Macherey-Nagel	Cat# 740412.10
Pierce™ BCA Protein Assay Kit	Thermo Fisher Scientific	Cat# 23227
DNeasy® Blood & Tissue Kit	QIAGEN	Cat# 69504
SuperSignal™ West Dura Extended Duration Substrate	Thermo Fisher Scientific	Cat# 34075

**Experimental models: Cell lines**		

BG1 ovarian cancer cell line	Oncotest GmbH	RRID: CVCL_6570

**Oligonucleotides**		

sgCDH1_F1: ACA CCG AGG CGG CGA GGA CGA CTA GG	This paper	N/A
sgCDH1_R1: AAA ACC TAG TCG TCC TCG CCG CCT CG	This paper	N/A
CDH1_PCR_F1: AGC CCC ACT CTG ATC TAT GG	This paper	N/A
CDH1_PCR_R1: GGG ACA CAC CAG TGT AGT AAT GA	This paper	N/A
EGFP-C_F: CAT GGT CCT GCT GGA GTT CGT G	This paper	N/A
EGFP-N_R: CGT CGC CGT CCA GCT CAG CAG	This paper	N/A
EGFP no IRES_F: ATG GTG AGC AAG GGC GAG	This paper	N/A
EGFP no IRES_R: CAA TTG CCG GTG ATG CGG	This paper	N/A
human_U6_F primer GAG GGC CTA TTT CCC ATG ATT	This paper	N/A

**Software and algorithms**		

FlowJo v10	Becton Dickinson	https://www.flowjo.com/
OMERO v5.6	OME project	https://www.openmicroscopy.org/omero
Adobe Illustrator	Adobe	https://www.adobe.com/ch_de/?mv=search&sdid=88X75SKX
Benchling	Benchling	https://benchling.com
NEBaseChanger	New England Biolabs	https://nebasechanger.neb.com/
4Peaks	Nucleobytes	https://nucleobytes.com/4peaks/

**Other**		

ViaFect™ Transfection Reagent	Promega	Cat# E4981
Precision Plus Protein Dual Color Standards	Bio-Rad	Cat# 1610374
T4 DNA Ligase	Promega	Cat# M180B
2× Rapid Ligation Buffer	Promega	Cat# C671B
RPMI-1640 Medium	Sigma	Cat# R8758
Fetal bovine serum	Sigma	Cat# F7524
Penicillin-Streptomycin	Sigma	Cat# P0781
Trypsin-EDTA solution	Sigma	Cat# T4174
Dulbecco’s Phosphate Buffered Saline	Sigma	Cat# D8537
RIPA Buffer (10×)	Cell Signaling Technology	Cat# 9806S
Protease Inhibitor Cocktail	Sigma	Cat# P8340
NEBuffer™ r3.1	New England Biolabs	Cat# B6003S
*BsmBI*-v2	New England Biolabs	Cat# R0739S
MyTaq™ DNA Polymerase	Bioline	Cat# BIO-21106
6× Gelladepuffer (Glycerin, Orange G)	Carl Roth	Cat# HP04.1
Immun-Blot® PVDF Membranes for Protein Blotting	Bio-Rad	Cat# 1620177
Whatman™ Chromatography paper	GE Healthcare	Cat# 3030-335
DNA-Marker pUC19/*Msp* I	Carl Roth	Cat# T149.1
1 kbp-DNA Ladder	Carl Roth	Cat# Y014.2
RedSafe™ Nucleic Acid Staining Solution (20.000×)	iNtRON Biotechnology	Cat# 21141
8-well, on glass slide, detachable	Sarstedt	Cat# 94.6170.802
CytoFLEX	Beckman Coulter	
BD FACSMelody™ Cell Sorter	BD Biosciences	
Nikon CSU-W1 plate loader	Nikon	

∗The vector is discontinued at Addgene. If not available in the laboratory, an alternative transfection control expressing EGFP under the control of a CMV promoter is also suitable.

## Materials and equipment

• LB Agar plates: add 10.5 g of LB Agar (Lennox) in 300 mL of ddH_2_O.

Autoclave the solution and store at 20°C–22°C. To prepare the plates, heat it up in a microwave and add the appropriate antibiotics. Store the plates at 4°C for up to 1 month.

• LB Broth: add 10 g of LB Broth (Lennox) in 500 mL of ddH_2_O.

Autoclave the solution and store at 20°C–22°C. As soon as antibiotics have been added store at 4°C for up to 1 month.

• 2×YT Broth: add 15.5 g of 2×YT Broth in 500 mL of ddH_2_O.

Autoclave the solution and store at 20°C–22°C for up to 3 months.

• Bromophenol Blue 10% solution: add 1 g of Bromophenol Blue in 10 mL of ddH_2_O.

Store at 20°C–22°C for up to 12 months.

• 2M DTT: add 3.085 g of DL-Dithiothreitol in 10 mL ddH_2_O.

Store at -20°C for up to 12 months.

• 10% SDS: add 1 g of SDS in 10 mL of ddH_2_O.

Store at 20°C–22°C for up to 12 months.

• 10% APS: add 1 g of Ammonium persulfate in 10 mL of ddH_2_O.

Store at 4°C for up to 12 months.

• Antibody dilution buffer: Prepare a solution of PBS containing 1% BSA and 0.1% Triton-X100, filter (0.2 μm).

Store at 4°C for up to 5 weeks.

• Blocking buffer: Prepare Antibody dilution buffer and add 5% FBS, filter (0.2 μm).

Store at 4°C for up to 5 weeks.

• Permeabilization buffer: Prepare a solution of PBS containing 0.25% Triton-X100.
  Store at 20°C–22°C for up to 3 months.
• Washing buffer: Prepare a solution of PBS containing 0.1% Tween 20.

Store at 20°C–22°C for up to 3 months.

**Table:** 10× Running Buffer

Reagent	Final concentration	Amount
Tris-Base	250 mM	30.3 g
Glycin	1.9 M	144 g
SDS	1% w/v	10 g
ddH_2_O	N/A	up to 1 L

Store at 20°C–22°C for up to 3 months.

**Table:** 10× Transfer Buffer

Reagent	Final concentration	Amount
Tris-Base	250 mM	30 g
Glycin	1.9 M	144 g
ddH_2_O	N/A	up to 1 L

Store at 20°C–22°C for up to 3 months.

**Table:** 10× TBS Buffer, pH 7.8

Reagent	Final concentration	Amount
Tris-Base	200 mM	24.2 g
NaCl	1.5 M	87.7 g
conc. HCl	N/A	∼12 mL
ddH_2_O	N/A	up to 1 L

Store at 20°C–22°C for up to 3 months.

**Table:** 4× Gel Loading Buffer

Reagent	Final concentration	Amount
Trizma hydrochloride	200 mM	1.58 g
SDS	8%	4 g
Glycerol	40%	20 mL
Bromophenol Blue	0.2%	100 mg∗
ddH_2_O	N/A	up to 50 mL

∗ Add 800 μL of a prepared 10% solution.

Store at -20°C for up to 12 months.

**Table:** 1.5 M Tris-HCl pH 8.8

Reagent	Final concentration	Amount
Trizma base	1.5 M	27.23 g
ddH_2_O	N/A	80 mL
Adjust to pH 8.8 with HCl	N/A	
ddH_2_O	N/A	up to 150 mL

Store at 20°C–22°C for up to 12 months.

**Table:** 1.0 M Tris-HCl pH 6.8

Reagent	Final concentration	Amount
Trizma base	1.0 M	12.11 g
ddH_2_O	N/A	60 mL
Adjust to pH 6.8 with HCl	N/A	
ddH_2_O	N/A	up to 100 mL

Store at 20°C–22°C for up to 12 months.

## Step-by-step method details

### Major step one: Generation of an E-cadherin EGFP knock-in ovarian cancer cell line: Delivery and gene-editing in cancer cells

**Timing: 2 days (for steps 1 and 2)**

**Figure 5:**
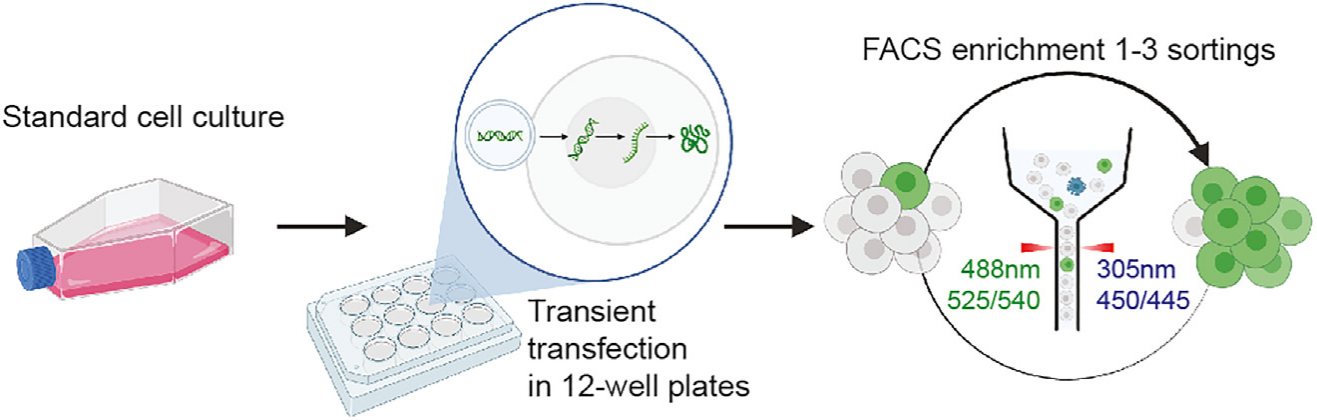
A schematic flowchart highlighting the key steps from cell culture to flow cytometry

The following describes the procedure to obtain a cancer cell line with functional E-cadherin knock-in. For representative purposes, we have utilized the human ovarian cancer cell line BG1 previously described to express sufficient amounts of E-cadherin.^1^ We describe the required cell culture conditions, cell line transfection using appropriate controls, and evaluation of knock-in cells using either fluorescence-activated cell sorting (FACS) or flow cytometry for EGFP^+^ cell sorting or analysis, respectively. A schematic flowchart for the knock-in procedure is shown in Figure 5.

1. Seeding of the BG1 ovarian cancer cell line.
  a. Grow the cells in a T75 culture flask using RPMI supplemented with 10% FBS and 1% Penicillin-Streptomycin.
  b. After reaching 80% confluence, harvest the cells using 1× Trypsin-EDTA (10× Trypsin-EDTA diluted 1:10 with PBS).
  c. Count the cells using either an automated cell counter or manual Neubauer counting chamber and prepare a cell suspension of 1 million cells per mL.
  d. Seed the cells at a density of 150′000 cells per well into a 12-well plate.
    i. Prepare a 12-well plate by adding 1 mL of culture media containing 150′000 cells per well.
    ii. Gently homogeneously distribute the cells in each well by manual horizontal shaking.
2. One day after cell seeding, prepare the plasmid combinations and perform the transfection.
  a. Prepare the plasmid pools and appropriate controls in 1.5 mL tubes as suggested in Table 8.

**Table 8:** Transfections (amounts in ng)

Plasmid	Sample	Controls			
	Knock-in	dCas9 (D10A)	Transfection efficiency	Double-cut EGFP only	Untreated
hCas9	600	–	–	–	–
hCas9_D10A		600	–	–	–
Sg-A	200	200	–	–	–
Double-cut EGFP only	600	600	–	600	–
sgCDH1	200	200	–	–	–
Transfection control	–	–	600	–	–
  b. Transient transfection of cancer cells.
    i. Exchange the culture medium of the cells at least 1–3 h before transfection using 1 mL of culture medium.
    ii. Add 100 μL of serum-free RPMI to each plasmid pool and vortex gently.
    iii. Pre-warm the ViaFect reagent to 20°C–22°C and mix by inverting it.
    iv. Add 4 μL of ViaFect to each plasmid pool, vortex gently, and spin down briefly.
    v. Incubate the reactions at 20°C–22°C for 20 min.
    vi. Add the reactions dropwise to the cells in the 12-well plate.
    vii. Slightly rock the plate to distribute the DNA-ViaFect, then incubate the cells at 37°C and 5% CO_2_ until the next day (troubleshooting 3).

**CRITICAL:** Be sure to use an early passage of your cell line of interest as the entire process of the knock-in generation will include multiple passaging steps.

A high concentration of plasmids is of benefit but for the transfection, they should be diluted allowing to pipet at least 1 μL to increase accuracy.

It is important to add appropriate controls to the transfection setup in order to know the overall efficiency of the transfection reagent in combination with the cell line of interest (e.g., pmaxGFP or pEGFP-N1) as well as the successful cleavage by the Cas9 (by comparing to the mutant hCas9_D10A) and, finally, to assess the signal generated by double-cut EGFP only alone. A non-transfected control should also be included.

The plasmid amounts provided in the table above are specifically for a 12-well format. Consequently, the amounts must be adapted if the format is changed but we suggest keeping the ratio of the plasmids.

### Major step two: Flow cytometry analysis and FACS enrichment of EGFP^+^ cells

**Timing: 4–7 weeks (for steps 3 to 5)**

After transfection, incubate the cells for 48 h or until they reach ∼80% confluency and perform analysis by flow cytometry in order to determine the percentage of EGFP^+^ cells together with the transfection efficiency (control plasmid). The percentage of the knock-in population might be very low at this stage. After keeping them in culture for up to two weeks, enrich the transfected cells for the EGFP^+^ population by fluorescence-activated cell sorting (FACS). Keep the EGFP^+^ cells under appropriate culture conditions until a sufficient cell number is reached for re-analysis. In order to achieve >80% EGFP^+^ cells, at least 2 to 3 FACS sorts are necessary. The procedure is explained in detail below.

**Figure 6:**
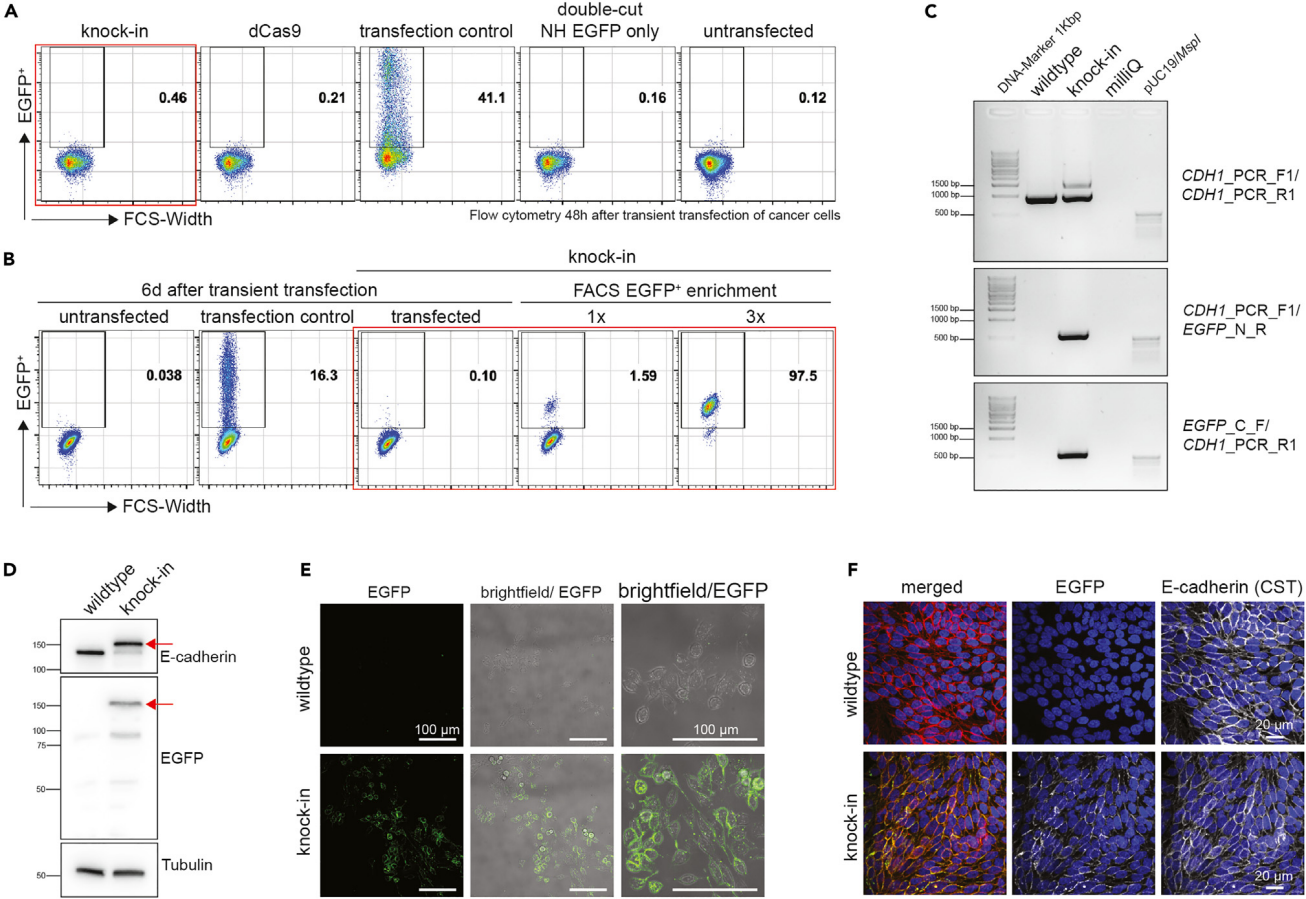
Expected outcome after integration of the EGFP reporter at the C-terminal E-cadherin encoding genomic locus (A) Flow cytometry 48 h after transfection, red box highlights the knock-in sample. Note: There might be some differences in gating cell populations as we used different CytoFLEX flow cytometers throughout the entire experiment. (B) Representative flow cytometry of cancer cells 6 days after transfection and after 1 and 3 rounds of FACS enrichment, red box highlights the knock-in sample. (C) Genotyping PCR showing successful integration of EGFP: The expected band at 954 bp indicates the wildtype whereas the additional band at 1712 bp (only visible in the knock-in sample) indicates successful knock-in. *In silico* analysis for second and third PCR should reveal only one band in the knock-in sample, CDH1_PCR_F1/EGFP_N_R 572 bp and EGFP_C_F/CDH1_PCR_R1 554 bp. (D) Representative Western blot for wildtype and knock-in cells harboring the EGFP-encoding DNA sequence indicated by the red arrow. (E) Confocal live cell imaging showing endogenous E-cadherin preferentially localized at the cell membrane. (F) Immunofluorescence of knock-in cells stained with antibody to E-cadherin shows colocalization of the EGFP and E-cadherin, DAPI nuclear staining, antibody to E-cadherin, and EGFP.

1. Preparing cells for flow cytometry analysis.
  **CRITICAL:** This should be done earliest 48 hours after transfection or when the cells reach ∼80% confluence. This initial analysis is crucial to determine the transfection efficiency, i.e. the percentage of EGFP^+^ cells in the ‘transfection efficiency’ control.
  a. Remove the culture medium and wash the cells by adding 300 μL of 1× PBS per well.
  b. Remove the PBS and add 300 μL of 1× Trypsin-EDTA.
  c. Incubate cells at 37°C and 5% CO_2_ until they completely detach from the plate (up to 15 min in the case of BG1).
  d. Add 600 μL of culture medium to stop the Trypsin reaction.
  e. Gently pipet the cells up and down to detach all remaining cells and to dissolve clumps.
  f. Place 300 μL of the suspensions into 1.5 mL Eppendorf tubes (for flow cytometry analysis) and plate the remaining 600 μL into a new 6-well plate.
    ***Note:*** The aim is to expand the knock-in cells (not the controls) at least to a T25 or a T75 flask for FACS (described in the next step). The controls can be kept for additional 2–3 passages and re-analyzed by flow cytometry at each passage to determine whether the GFP signal is decreasing.
  g. Centrifuge the Eppendorf tubes for flow cytometry analysis at 400 × *g* for 5 min at 20°C–22°C and resuspend in 120 μL of flow cytometry buffer (1× PBS containing 1% of FBS).
  h. Transfer the cell suspensions to a 96-well plate and proceed to flow cytometry analysis, in our case by CytoFLEX (Beckman Coulter, USA) (Figure 6A).

2. Preparing cells for FACS.
  ***Note:*** The cells should have reached 80% confluence of a T25 or a T75 flask. The following protocol considers a T75 flask.
  a. Remove the culture medium and wash the cells by adding 3 mL of 1× PBS.
  b. Remove the PBS and add 3 mL of 1× Trypsin-EDTA and incubate at 37°C and 5% CO_2_ until completely detached from the flask.
  c. Add 6 mL of culture medium to stop the Trypsin reaction, gently rinse the flask by pipetting and transfer the cells to a 15 mL tube.
  d. Remove 0.5 mL of the suspension and plate it in a T25 flask to keep a backup of the cells in case problems occur during or after the sorting. Centrifuge the remaining cells at 200 × *g* for 5 min at 20°C–22°C, and remove the supernatant.
    ***Optional:*** DAPI staining for live/dead distinction during FACS: Resuspend the cells in 200 μL of DAPI solution (DAPI diluted 1:5000 in flow cytometry buffer) and incubate for 5 min at 20°C–22°C in the dark. Centrifuge at 200 × *g* for 5 min at 20°C–22°C, remove the supernatant and resuspend in 1 mL of flow cytometry buffer. Repeat this washing step twice more. Remove the supernatant.
  e. Resuspend the cells in 1 mL of flow cytometry buffer and pass through a FACS tube with a cell strainer cap (70 μm).
  f. Prepare a second tube containing culture media which will be used to collect the sorted cells.
    ***Note:*** The collection tube size should be adapted to the number of cells expected to be positive.
  g. Proceed to the sorting.
  h. Centrifuge the sorted cells at 400 × *g* for 5 min at 20°C–22°C, remove the supernatant very carefully and leave ∼300–500 μL of liquid in the tube.
  i. Resuspend the cells and plate them into a well.
  j. Add an appropriate volume of fresh culture medium on top (troubleshooting 4 and 5).
    ***Note:*** The well size should correspond to the number of collected cells, e.g. around 1000 cells can be plated in a 48- or 24-well plate depending on the doubling time of the cell line (in our case, BG1 is a fast-growing cell line).

3. Further procedure:
  a. Exchange the medium one day after sorting.
  b. Expand the cell culture until reaching a sufficient number of cells (25cm^2^ culture flask with ∼3 × 10^6^ live cells) for flow cytometry analysis to determine the EGFP^+^ enriched population and repeat step 3 (Preparing cells for flow cytometry analysis) (Figure 6B).
  c. Repeat step 4 (Preparing cells for FACS) if another enrichment is necessary. These enrichment cycles should be repeated until the desired percentage of EGFP^+^ cells is reached (Figure 6B).
  d. As soon as the cells have been sufficiently enriched, expand them and either proceed to your individual experiments or freeze them using freezing medium (FBS containing 10% DMSO, filtered 0.2 μm).

**Pause point:** Cells can be frozen at this point and are stable at −80°C for up to 12 months or several years when stored in liquid nitrogen.

**CRITICAL:** It is crucial to choose an appropriate well size to plate the cells after sorting. The cells will not grow well or take more time to reach full confluence if the chosen well size is too large. The doubling time of the cell line should also be considered.

### Major step three: Validation of the *CDH1* knock-in cells by PCR, Western blot, and immunofluorescence

**Timing: 1 week (this is flexible as not everything has to be performed at the same time) (for steps 6 to 8)**

The successful integration of the EGFP-encoding DNA sequence at the C-terminus of E-cadherin must be further validated, in addition to the confirmation by flow cytometry analysis. Here, we address genomic DNA and the expression of the tagged protein.

1. Genomic DNA isolation and genotyping PCR:
  a. Harvest the EGFP^+^ enriched cell line using 1× Trypsin-EDTA as usual and take ∼10^6^ cells in a 1.5 mL tube and centrifuge at 400 × *g* for 5 min at 20°C–22°C.
  b. Remove the medium and resuspend in 1 mL of 1× PBS.
  c. Centrifuge at 400 × *g* for 5 min at 20°C–22°C and remove the supernatant.
    **Pause point:** The cell pellet can be stored at -20°C until further processed.
  d. Perform genomic DNA isolation with the DNeasy® Blood & Tissue Kit following the manufacturer’s protocol (LINK).
    ***Note:*** Concerning the elution, 25–50 μL of the provided elution buffer is usually sufficient depending on the number of cells.
  e. Measure the concentration and quality of the extracted genomic DNA by Nanodrop.
    ***Note:*** Concentrations above 200 ng/μL, A260/280–1.8 and A260/230 2.0–2.2 can be considered pure and of good quality.
  f. Dilute the genomic DNA to 100 ng/μL.
  g. Prepare the PCR reaction master mix following Table 9 below. Please note that the volumes given are for one reaction. These should be multiplied by the total amount of reactions needed. In our case, we prepared the master mix for four reactions (BG1 wildtype, BG1 CDH1-EGFP knock-in, water control, and one additional reaction to compensate for pipetting errors). In addition, three independent master mixes should be prepared, one for each primer pair: CDH1_PCR_F1 / CDH1_PCR_R1, CDH1_PCR_F1 / EGFP-N_R, EGFP-C_F / CDH1_PCR_R1. Prepare the master mix in 1.5 mL tubes without adding the DNA. Pipet 9 μL of the master mix into four PCR tubes and add the respective DNA or ddH_2_O to each tube. Mix the tubes gently and spin them down briefly.

**Table 9:** PCR reaction master mix

Reagent	Amount per reaction
DNA template	100 ng
MyTaq DNA Polymerase (5U/μL)	0.1 μL
Forward primer (10 μM)	0.2 μL
Reverse primer (10 μM)	0.2 μL
5× MyTaq Reaction Buffer	2 μL
ddH_2_O	up to 10 μL
  h. Place the PCR tubes into the thermocycler and apply the following programme (Table 10):

**Table 10:** PCR cycling conditions

Steps	Temperature	Time	Cycles
Initial Denaturation	95°C	5 min	1
Denaturation	95°C	15 s	35 cycles
Annealing	58°C	15 s	
Extension	72°C	2 min	
Final extension	72°C	5 min	1
Hold	16°C	Forever
  i. In the meantime, prepare a 1.2% (w/v) agarose gel.
  j. When the PCR reaction is finished, add 2 μL of 6× Loading dye to each sample, mix and spin down briefly.
  k. Load everything onto the agarose gel and let it run at 120 V for ∼20–30 min or until a sufficient separation has been achieved.
  l. Take an image of the gel using a gel doc system (Figure 6C).
    **CRITICAL:** It is important to start the genomic DNA extraction with a sufficient number of cells in order to obtain high-quality DNA with a sufficient yield.
    It is also important to adapt the PCR master mix if you are using a different polymerase and to change the PCR conditions and percentage of agarose gel depending on the expected size of your amplicon. If the wildtype and knock-in bands only show a small difference in size, you should also increase the time of gel electrophoresis for better separation.

2. Preparation of protein lysates for Western blot analysis.
  a. Preparation of protein lysates:
    i. Culture the cells in a petri dish (10 cm diameter) until reaching 80%–90% confluence.
    ii. Remove the culture medium and wash the cells with 5 mL of cold 1× PBS.
    iii. Completely remove the PBS and add 350 μL of 1× RIPA (dilute 10× RIPA with ddH_2_O) containing 1:100 Protease Inhibitor Cocktail, i.e., 3.5 μL.
    iv. Immediately place the dish on ice and incubate for 5 min.
    v. Harvest the cells with a cell scraper and transfer the cell suspension into a 1.5 mL tube.
    vi. Incubate on ice for 20 min.
    vii. Centrifuge full speed at 4°C for 20 min.
    viii. Transfer the supernatant into a new 1.5 mL tube.
      ***Note:*** From now on, always keep the protein lysates on ice.
      **Pause point:** The protein lysates can be stored at -80°C for long-term storage. Thaw on ice for further usage.
    ix. Measure the protein concentration with Pierce™ BCA Protein Assay Kit according to the manufacturer’s protocol (LINK).
    x. Dilute the protein lysates to a concentration of 2 μg/μL using ddH_2_O and add 4× gel loading buffer (incl. DTT: add 100 μL of 2M DTT solution to 400 μL of 4× gel loading buffer).
    xi. Boil at 95°C for 5 min to denature the proteins. From this point on, the samples can be handled at 20°C–22°C. The boiled lysates can be loaded onto an acrylamide gel or stored at −20°C.
      **Pause point:** The boiled protein lysates can be stored at −20°C for long-term storage. After thawing, heat them up for 2 min at 60°C. They are now ready to use.
  b. Preparation of 10% acrylamide/bis gels for Western blot.
    ***Note:*** This should be done inside a chemical hood.
    i. Clean the glass plates (Bio-Rad, thickness 1.5 mm) with ddH_2_O, then with 70% ethanol and assemble them according to the manufacturer.
    ii. Prepare the resolving gel in a 50 mL tube combining the following reagents in the given order (consider 10 mL per gel) in Table 11:
      ***Note:*** As soon as the TEMED has been added, pour the gel immediately as the polymerization will begin.

**Table 11:** Resolving gel

Reagent	Amount (in mL)
ddH_2_O	4.0
30% Acrylamide/Bis mix	3.3
1.5 M Tris (pH 8.8)	2.5
10% SDS	0.1
10% APS	0.1
TEMED	0.004
**Total**	**10 mL**
    iii. Pour the gel between the assembled glass plates leaving enough space for the stacking gel (∼1.5 cm from the top).
    iv. Add a layer of isopropanol or ddH_2_O on top and leave the gel to solidify for ∼45 min.
    v. Prepare the stacking gel in a 50 mL tube combining the following reagents in the given order (consider 3 mL per gel) in Table 12:
      ***Note:*** As soon as the TEMED has been added, pour the gel immediately as the polymerization will begin.

**Table 12:** Stacking gel

Reagent	Amount (in mL)
ddH_2_O	2.1
30% Acrylamide/Bis mix	0.5
1.0 M Tris (pH 6.8)	0.38
10% SDS	0.03
10% APS	0.03
TEMED	0.003
**Total**	**3 mL**
    vi. Pour off the layer of isopropanol or ddH_2_O and pour the stacking gel by filling the glass plates completely to the top.
    vii. Immediately add the comb (in our case with 10 wells) which must be suitable for 1.5 mm glass plates.
    viii. Leave the gel to solidify for ∼15 min.
      **Pause point:** The gels can now be wrapped in moist tissue paper and kept in a sealed plastic bag at 4°C for up to 1 week.
  c. Assembly and SDS-PAGE.
    i. Prepare 1× Running Buffer: Dilute 10× Running Buffer by adding 100 mL–900 mL of ddH_2_O.
    ii. Remove the glass plates with the gels from the Bio-Rad system, remove the combs carefully and rinse the glass plates and the wells with ddH_2_O.
    iii. Assemble the glass plates with the gels inside the electrophoresis apparatus and fill up the middle with 1× Running Buffer until reaching the top of the glass plates. Fill up the outside of the glass plates as far as indicated on the chamber.
    iv. Load 5 μL of the ladder and the desired amount of the protein lysates into the wells.
    v. Close the electrophoresis apparatus and apply 90 V until the samples reach the resolving gel, then increase to 120 V until a good separation is achieved. This can be verified by following the separation of the ladder.
      ***Note:*** At some point, the smallest proteins will leak out of the bottom of the gel and be lost, so stop it in time.
  d. Transfer onto PVDF membrane.
    i. Prepare 1× Transfer buffer as described in the following table and place it at 4°C. It can be reused up to 3 times.

**Table:** 

Reagent	Amount (in mL)
10× Transfer Buffer	100 mL
ddH_2_O	700 mL
100% Methanol	200 mL
**Total**	**1 L**
    ii. Soak a PVDF membrane in methanol inside a glass petri dish for 30 s, then transfer it to ddH_2_O.
    iii. Soak sponges and Whatman paper in a container with 1× Transfer buffer.
    iv. Remove the glass plates from the electrophoresis apparatus, separate the plates and place the gel into the container with 1× Transfer buffer.
    v. Assemble the sandwich consisting of a sponge, Whatman paper, gel, PVDF membrane, Whatman paper, and sponge and place it inside the transfer apparatus.
    vi. Fill the chamber with 1× Transfer buffer and add a container with ice.
    vii. Apply 75 V for 1.5 h in total. Exchange the ice cuvette after 45 min.
      ***Note:*** The transfer time might have to be adapted depending on the protein size. For large proteins, it might be necessary to exchange the ice again and add e.g. 30 min of transfer time.
      ***Alternatives:*** Nitrocellulose can also be used instead of PVDF membranes.
  e. Blocking:
    i. Prepare 1× TBST: Dilute 50 mL of 10× TBS in 450 mL ddH_2_O and add 500 μL of Tween 20.
    ii. Prepare 1× TBST with 3% BSA, e.g., weigh 1.5 g and dissolve in 50 mL of 1× TBST by placing it on a roller shaker at 20°C–22°C.
    iii. Place the PVDF membrane in a small container with 20 mL of 1× TBST with 3% BSA.
    iv. Keep it at 20°C–22°C for at least 30 min with gentle agitation.
  f. Primary antibodies.
    i. Prepare the primary antibody solutions in 50 mL tubes by taking 5 mL of 1× TBST with 3% BSA and adding the desired primary antibody following the manufacturer’s recommended dilution for Western blot.
    ii. Prepare a clean surface and place the PVDF membrane on top.
    iii. Cut it at the desired size (use the ladder bands to know where to cut).
    iv. Place the membrane pieces in the corresponding primary antibody containing 50 mL tubes.
    v. Incubate at 4°C for 16 h overnight on a roller shaker.
    vi. Remove the membrane pieces from the tubes and place them in individual boxes.
      ***Note:*** The tubes containing the primary antibody solution can be frozen at −20°C and reused several times.
    vii. Add 5 mL of 1× TBST to each membrane piece and incubate for 10 min with gentle agitation at 20°C–22°C. Then discard the solution. Repeat this washing step twice more.
  g. Secondary antibodies.
    i. Prepare the secondary antibody solution by diluting the antibody 1:10′000 in 1× TBST (e.g., 10 mL containing 1 μL of secondary antibody).
    ii. Add 5–10 mL of the secondary antibody solution to each membrane piece and incubate for 3 h at 20°C–22°C with gentle agitation. Then discard the secondary antibody solution (do not reuse).
    iii. Add 5 mL of 1× TBST to each membrane piece and incubate for 10 min with gentle agitation at 20°C–22°C. Then discard the solution. Repeat this step twice more.
  h. Development.
    i. Place the membrane on a clean surface, e.g., a piece of plastic foil which has been cleaned with 70% ethanol and drain excess liquid with a tissue (avoid touching the surface of the membrane with the tissue).
    ii. Mix equal amounts of SuperSignal™ West Dura Luminol/Enhancer and SuperSignal™ West Dura Stable Peroxide from the kit SuperSignal™ West Dura Extended Duration Substrate and distribute dropwise onto the membrane until the whole surface is covered.
    iii. Perform the measurement at a gel doc, in our case a Vilber Fusion FX, according to the manufacturer and save a digital image of the membrane (Figure 6D)**.**
      ***Note:*** The exposure time depends on the expression/abundance of the protein of interest and must be optimized.

3. Immunofluorescence imaging.
  a. Cell culture.
    i. Culture the cells until reaching 80% confluence.
      ***Note:*** Independent of the confluency, knock-in cells can be visualized by fluorescence microscopy anytime using brightfield and EGFP channel (Figure 6E). In order to increase the quality of fluorescence imaging, we recommend culturing cells on microscopy-compatible culture dishes (e.g. Sarstedt, #94.6170.802).
    ii. Harvest the cells using 1× Trypsin-EDTA as usual and count the cells using either an automated cell counter or manual Neubauer counting chamber.
    iii. Add 20′000–50′000 cells in 500 μL of culture medium into each well of an 8-well glass chamber slide (detachable, Sarstedt, #94.6170.802).
    iv. Pipet up and down to distribute the cells homogeneously.
    v. Culture the slide inside a petri dish (10 cm diameter; containing a wet tissue to avoid evaporation of the medium) for 2 days at 37°C and 5% CO_2_ or until the cells reach 80% confluence.
      ***Note:*** The desired confluence depends on the protein of interest which shall be visualized. A too-high or too-small density might influence the protein expression.
  b. Fixation, permeabilization, and blocking.
    i. Carefully remove the culture medium and wash once by adding 300 μL of 1× PBS.
      ***Note:*** All the pipetting steps must be done very carefully as the cells might otherwise be detached quite easily from the slide.
    ii. Fix the cells by adding 150 μL of 4% formalin solution carefully to the cells and incubate for 10 min at 20°C–22°C.
      ***Note:*** Handling 4% formalin solution should always be done using a chemical hood. This includes the following washing steps until being sure that all remaining formalin is removed.
    iii. Carefully remove the formalin solution and add 500 μL of 1× PBS to wash. Repeat this washing step twice more.
      **Pause point:** At this step, the slide can be kept at 4°C for up to 2 weeks but the cells must be covered well with PBS to avoid drying out.
    iv. Permeabilize the cells by adding the permeabilization buffer and incubate for 5 min at 20°C–22°C. Avoid a longer incubation time.
    v. Remove the permeabilization buffer and wash once with 500 μL of 1× PBS.
    vi. Remove the 1× PBS and add 200 μL of blocking buffer.
    vii. Incubate at least for 1 h at 20°C–22°C.
      ***Optional:*** The blocking can also be done overnight at 4°C.
    viii. Prepare the primary antibody solution by adding the primary antibody to the antibody dilution buffer in an appropriate dilution for immunofluorescence suggested by the manufacturer.
    ix. Remove the blocking buffer and add 250 μL of the primary antibody solution and incubate 16 h overnight at 4°C.
  c. Staining and mounting.
    i. Remove the primary antibody solution and add 500 μL of washing buffer. Remove the washing buffer and repeat this washing step twice more.
    ii. Prepare the secondary antibody solution by diluting the secondary antibody 1:500 in antibody dilution buffer and add 250 μL of the secondary antibody solution to the cells.
    iii. Incubate at least for 3 h in the dark at 20°C–22°C.
    iv. Remove the secondary antibody solution and carefully remove the upper part of the slide (starting from one edge). Try to do this in a slow and steady movement to avoid breaking the cell layer.
    v. Wash the slide by immersion into 1× PBS in a light-protected container.
    vi. Remove any excess liquid from the slide by gently tapping the edge of the slide onto tissue paper.
    vii. Add 150–200 μL of ProLong Gold mounting media (containing DAPI) in the middle of the slide.
    viii. Carefully cover the cells with a 24 mm × 60 mm coverslip.
      ***Note:*** Avoid trapping any air bubbles under the coverslip as later the imaging will not be possible at this spot.
    ix. Seal the edges of the coverslip with transparent nail polish and allow the mounting medium to cure for 5 min in the dark.
      **Pause point:** The slide can be stored at 4°C in the dark.
    x. Allow the slide to cure for 1 day at 4°C in the dark.
    xi. Proceed to a (confocal) fluorescence microscope and perform the analysis. In our case, we used a spinning-disk Nikon CSU W1 confocal microscope (CFI Plan Apo Lambda 40× Air NA0.95) and images were stored in the secure Open Microscopy Environment OMERO for reproducible and robust image analysis (University of Basel, Imaging core facility) (Figure 6F).

## Expected outcomes

The current protocol allows us to C-terminally tag the transmembrane glycoprotein E-cadherin in the ovarian cancer cell line BG1 using the CRISPR-Cas9 system and NHEJ. A set of validation methods confirms successful integration of the EGFP encoding sequence (Figure 6). The tagged protein can theoretically be used for downstream assays studying factors impacting CDH1 protein expression, to immuno precipitate the protein via the EGFP tag to further characterize the protein in regards to post-translational modifications or potential binding partners, or to trace endogenous E-cadherin expressing cells *in vitro* and *in vivo*. In principle, the method described herein allows us to tag any protein of interest by designing a new sgRNA targeting a desired genomic protein-encoding locus. In the meantime, we have applied this experimental procedure to breast cancer (MCF-7 and T47D) and leukemia (HEL) cell lines tagging either E-cadherin or CD117 encoded by *KIT*, respectively (data not shown).

## Limitations

Generation of knock-in cells using fluorescence proteins such as the herein-described EGFP highly relies on the targeted cell lines. Here, the gene editing efficiency also depends on the transfection efficiency obtained with the commercially available transfection reagent. Another important aspect is the endogenous expression of the protein of interest. Low expression may result in insufficient fluorescence intensity for downstream enrichment and analysis. Thus, the protein expression should be determined in advance.

Another important factor is the genomic locus for insertion which can obviously cause frameshift mutations or the integration of the EGFP-encoding DNA sequence may interfere with the function of the protein as highlighted in Cumin et al. 2022.^1^

In case FACS enrichment is not a valid option, it is plausible to consider a selection of individual cell clones harboring the knock-in of interest. We believe that our described approach enriching for EGFP^+^ cells reduces the potential pitfall of clonal effects considering the heterogeneity of cancer cell lines. This is supported by single-cell transcriptomics of cancer cell lines which revealed that clinically relevant markers were maintained, however, authors show that breast cancer cell lines express these markers heterogeneously among cells even within the same cell line. Moreover, they observed dynamic plasticity in the regulation of HER2 expression in the MDA-MB-361 cell line with striking consequences on drug response.^10^ This reported finding may be an issue while working with selected clones. Apart from cancer cell line heterogeneity, avoiding clonal selection also massively reduces the costs considering that each individual clone derived from a parental knock-in culture requires characterization which is mandatory before further use. In addition, after delivering the plasmid set, single cells have to be isolated to generate clonal cells that can be verified as valid knock-ins. Here, limited dilution cloning and FACS are common approaches, but none is ideal in terms of efficacy, control of the selection and cell viability. Limiting dilution cloning is economical and may cause less cellular stress than FACS sorting, but it is severely limited in generating highly validated single-cell clones. Furthermore, the clonal selection is also introducing affinity-based selection steps^11^ which downstream may not represent the parental cell characteristics.

## Troubleshooting

### Problem 1

Potential re-cutting of Cas9 at the targeted genomic locus (related to Preparation 2).

### Potential solution

It is important to mention that our experimental setup in principle utilizes any sgRNA sequence together with the described plasmid set which we consider as an advantage over traditional CRISPR-Cas9 HDR strategies. However, our described experimental setup is limited to available sgRNA recognition sites at the desired genomic locus. Our method also does not allow mutation of the guide recognition site to minimize re-cutting by Cas9 endonuclease after successful gene-editing. We aim to overcome this potential limitation by transiently delivering our set of plasmids including the Cas9 encoding vector.

### Problem 2

Low *in silico* scores predicted by benchling (related to Preparation 2 / Step 6).

### Potential solution

In principle, benchling suggests to select sgRNA guides with high on-target and off-target scores. Our protocol relies on the genomic locus guide recognition site for in-frame insertion of the fluorescence protein. Here, a low on-target score may explain low percentage of EGFP^+^ cells. We considered the *in silico*-predicted on- and off-target score with less priority.

### Problem 3

Transfection of the human cells leads to increased cell death resulting in many floating/detached cells on the next day (related to Step 2).

### Potential solution

Before starting the actual experiment, it is recommended to test different transfection reagents with the cell line of interest using a control plasmid. This will provide information on which reagent is the most suitable resulting in viable cells and high transfection efficiency. Apart from lipofection, electroporation would be an alternative to enhance transfection efficiency.

### Problem 4

In general, FACS enrichment results in a low number of EGFP^+^ cells. This in turn may lead to insufficient growth (related to Step 4).

### Potential solution

Due to expected low knock-in efficiency resulting in a low number of EGFP^+^ cells, make sure to add enough culture medium in the FACS collection tube as cells might be lost if they stick to the wall of the tube.

Additionally, plate the cells in a smaller well size enabling closer proximity of the cells. This also depends on the cell line of interest which might be more or less capable of tolerating the stress of sorting and of being plated at low density.

### Problem 5

Low or non-detectable EGFP^+^ cells due to out-of-frame insertion (related to Step 4).

### Potential solution

Expression of E-cadherin C-terminally tagged with an out-of-frame fluorescence protein may show different molecular weight using Western blot analysis. Independently consider another sgRNA recognition site for insertion of the fluorescence protein.

## Resource availability

### Lead contact

Further information and requests for resources and reagents should be directed to and will be fulfilled by the lead contact [Francis Jacob] (francis.jacob@unibas.ch).

### Materials availability

All materials used in this experiment are available through commercial resources and addgene as highlighted in the key resources table with the exception of the modified double-cut EGFP only derived from the double cut NH-donor. The plasmid can be shared upon request to the lead contact, Francis Jacob (francis.jacob@unibas.ch).

## Data Availability

To the best of our knowledge, we have provided all information necessary. If any further information is required to perform the experiment described in this work, the lead contact welcomes requests.
